# Unveiling the dual-pathway effect of CSR perception environmental on employee pro-environmental behavior: evidence from Chinese marine enterprises

**DOI:** 10.3389/fpsyg.2025.1649048

**Published:** 2025-08-25

**Authors:** Xu Duan, Jiangyue Wu

**Affiliations:** ^1^College of Business Administration, Capital University of Economics and Business, Beijing, China; ^2^Marine Disaster Reduction Center, Ministry of Natural Resources, Beijing, China

**Keywords:** meaningful work, connectedness to nature, perception of environmental CSR, pro-environmental behavior (PEB), green psychological climate

## Abstract

To achieve the sustainable development goals and in response to the green development policies, many enterprises have actively incorporated corporate social responsibility (CSR) into their strategic plans in order to enhance environmental sustainability. This study explores the cognitive and emotional pathways through which perceived environmental CSR (PECSR) influences pro-environmental behavior (PEB) among employees in China’s marine enterprises, based on the Cognitive-Affective Personality System theory. The research was conducted through data collection and verified through the structural equation model. PECSR is significantly and positively associated with PEB, mediated by meaningful work and connectedness to nature, with a green psychological climate further supporting this relationship. Results highlight PECSR as a critical factor in CSR effectiveness. When planning sustainability strategies, enterprises should consider the significant impact of PECSR on employee behavior to foster environmentally responsible practices.

## 1 Introduction

Global sustainable development is at a critical juncture. The proportion of Sustainable Development Goals (SDGs) that have been achieved is only 15 percent, a figure that highlights the urgency of the environmental crisis (Thiermann et al., 2020; United Nations, 2023; Wang and Chen, 2024). A deteriorating global environment has led governments around the world to formulate policies and laws that promote green and sustainable development through various means (Deng et al., 2023a; Deng et al., 2023b; Deng et al., 2024; Mi et al., 2020; Xiao et al., 2024). This scenario indicates that the manufacturing sector is a sector that must become more environmentally friendly (Kasim, 2009; Liu et al., 2023; Nguyen, 2024; Rahman and Post, 2012). Its green transition is widely regarded as pivotal to achieving SDG 9 (Industry, Innovation and Infrastructure) and SDG 13 (Climate Action). As a result, various corporations have undertaken proactive measures to make their operational processes more environmentally friendly, integrating corporate social responsibility (CSR) into their strategic development plans (Barragán-Ocaña et al., 2023; Deng et al., 2022b; Lei et al., 2022; Vázquez-Carrasco and López-Pérez, 2013). Yet whether corporate green strategies actually materialize ultimately depends on micro-level employee behavior. Employees, as the final implementers, determine the effectiveness of energy-saving and emission-reduction initiatives through their daily pro-environmental behavior (PEB). In order to enhance employees’ awareness and literacy regarding environmental protection, the enterprise’s proactive behaviors in assuming social responsibility play an important and exemplary role. Employees’ awareness of environmental protection can be effectively stimulated by these behaviors. The importance of CSR cannot be denied (Madime and Gonçalves, 2022; Shah et al., 2021). Employee perception of CSR is determined by the extent to which they recognize the company’s endorsement of a variety of social initiatives (Lee et al., 2013). The comprehensive exploration of employee perceptions of CSR is critical to fostering a more profound understanding of the theoretical framework and the detail practices associated with it. This study explores the potential role of perceived environmental corporate social responsibility (PECSR) in fostering employee behaviors and attitudes, thus providing valuable insights into the dynamics of organizational behavior and employee wellbeing (Gond et al., 2023). There are three reasons we focus on Chinese marine enterprises. First, China’s marine economy surpassed 10 trillion RMB for the first time in 2024. However, marine ecological degradation etc., have become significant risks to its continued growth. Second, although CSR initiatives in the marine sector have proliferated, micro-level research on their effects remains scarce. Third, existing studies have predominantly focused on land-based industries, leaving the CSR impacts within the unique “marine context” largely unexplored.

This paper is structured as follows: the literature review is presented in Section 2, and the theoretical foundation and hypotheses are presented in Section 3. Section 4 describes the methodology. Section 5 presents the results of the descriptive and factorial analyses. Lastly, Sections 6 and 7 present the discussion and conclusions.

## 2 Literature review

The examination of CSR perceptions has been the subject of a substantial body of research, including identification of organizations, stakeholder groups, management, business models, investment, value, innovation, corporate strategies, media, national practices, causal attribution, and sustainable development (Bagire et al., 2011; Cheema et al., 2020; Chih, 2011; Fox et al., 2020; Frynas and Yamahaki, 2019; Glavas and Kelley, 2014; González-Rodríguez et al., 2013; Latapí Agudelo et al., 2019; Lei et al., 2022; Li, 2020; Liu M. et al., 2021; Lyra et al., 2017; Mackenzie and Peters, 2014; Murshed et al., 2023; Shauki, 2011; Skouloudis et al., 2011; Skouloudis et al., 2015; Smirnova, 2012; Taliento and Netti, 2020; Tench et al., 2007; Viveros, 2016; Wang and Juslin, 2011; Wang and Juslin, 2012; Windsor, 2001; Wong et al., 2010; Wood et al., 2021; Yan et al., 2023; Yuriev et al., 2018; Zubeltzu-Jaka et al., 2018). It further subdivides CSR perceptions into the social aspects of CSR and the environmental aspects of CSR as a mainstream concept for corporations to address their ethical, social, and environmental responsibilities toward society (Chwiłkowska-Kubala et al., 2021; Setiono and Hidayat, 2022; Stojanović et al., 2020; Wickert and Risi, 2019). Environmental perception from a consumer and related perspective is one of the main focus areas of CSR (Bardos et al., 2020; Becker-Olsen et al., 2006; Bigné et al., 2012; Di Vaio et al., 2020; Costa et al., 2020; Currás-Pérez et al., 2018; González-Rodríguez and Díaz-Fernández, 2020; Huang et al., 2019; Nurunnabi et al., 2018; Ramasamy and Yeung, 2009; Singh et al., 2008). In addition to generating ideas and promoting implementation of ideas (Yu and Wu, 2024), environmental corporate social responsibility (ECSR) (Li et al., 2022) practices have positive and significant effects on environmental well-being (EWB) in the local community (Rela et al., 2020). The value of ESCR depends on both market and non-market forces (Lyon and Maxwell, 2008). The green corporate image of an organization is a function of its employees’ involvement in ECSR activities as manifested by their environmental passion and PEB (Ali et al., 2023). PECSR is a psychological perception that employees have when implementing energy conservation, environmental protection, pollution control, and other policies in the production and operation processes of an enterprise (Li et al., 2022; Sarfraz, 2023; Wu et al., 2022).

Corporate Social Responsibility (CSR) can enhance employee loyalty and, as a result, improve enterprise performance (Jin et al., 2024). Employee service can be improved through CSR (Brown, 1990). Through organizational identification, employees’ perceptions of CSR indirectly affect their engagement in voluntary PEB, and these effects are stronger for employees who are highly empathic (Shah et al., 2021; Tian and Robertson, 2019). Moral efficacy strengthens the indirect effects of perceived CSR on voluntary PEB through organizational commitment and ethical leadership (Bai et al., 2024; Molnár et al., 2021). Employee perceptions of CSR are positively correlated with their attitudes and behaviors (Asante Boadi et al., 2020; Duarte and Mouro, 2022; Farrukh et al., 2020; Min et al., 2024), and negatively correlated with their negative attitudes and behaviors (Wang et al., 2020). The relationship between work meaningfulness and perceived organizational support is also positively related to organizational commitment (Glavas and Kelley, 2014). PEB is also positively influenced by employee perceptions of CSR (Liu et al., 2023).

Nevertheless, CSR skepticism weakens the link between perceived CSR and PEB, whereas CSR authenticity strengthens it (Liu et al., 2023). CSR activities drive organizational identification and encourage coworkers’ environmental advocacy, which in turn generates employees’ PEBs (Gkorezis and Petridou, 2017; Shah et al., 2021). The relationship between CSR and organizational citizenship behaviors toward the environment is mediated by organizational pride (Hameed et al., 2019; Han and Chen, 2024). CSR on environmental performance is also influenced by employees’ PEB (Ahmad et al., 2021). Employee PEB can be affected by perceived CSR in three ways: direct effect, indirect effect via environmental consciousness, and indirect effect via environmental commitment (Wang et al., 2023). In fact, the PECSR will influence employees’ behaviors (Yu and Wu, 2024). However, employees are passive receivers of CSR information, and little research has been done on how CSR practices influence their perception of the environment.

## 3 Theoretical foundation and hypotheses development

### 3.1 Theoretical foundation

Individual behavior is greatly influenced by psychological factors, which can effectively explain how behavior changes over time. Personality information is gathered not only through naturalistic means, but also through expert assessments (Kammrath and Scholer, 2012). In general, people use personality information for three purposes: prediction, explanation, and influence (Kammrath and Scholer, 2012). These purposes are met by Mischel and Shoda’s Cognitive Affective Personality System (CAPS) theory (John et al., 2008; Mischel and Shoda, 1995; Mischel and Shoda, 1998). While the Theory of Planned Behavior (TPB) emphasizes behavioral intention shaped by attitudes, subjective norms, and perceived control (Rhodes and Courneya, 2003), it offers limited insight into how affective reactions might moderate or conflict with cognitive evaluations of CSR, nor does it account for within-person variability in these processes. Social Exchange Theory (SET) and Organizational Identification (OI) focus on reciprocal benefits or self-definition (Van Knippenberg and Sleebos, 2006), thereby under-specifying the moment-to-moment intra-individual variability that arises when employees appraise CSR in real time. CAPS, by contrast, models idiosyncratic “if-then” signatures, where cognitive and affective units are dynamically activated by CSR-related situational cues, providing a finer-grained lens for explaining heterogeneous employee responses. In the field of personality psychology, the CAPS theory introduced an important new paradigm (Metcalfe and Mischel, 1999). Several theories of more specific personality patterns fall within the scope of CAPS as a meta-theory of personality (John et al., 2008; Metcalfe and Mischel, 1999; Mischel and Shoda, 1998). Numerous aspects of participants’ CAPS dynamics have been examined by researchers using multilevel modeling (Bolger and Zuckerman, 1995; Côté and Moskowitz, 1998; Fleeson, 2007; Kammrath et al., 2015; Kammrath, 2011; Rhodewalt and Morf, 1998; Suls and Martin, 2005; Wood and Brumbaugh, 2009; Zautra et al., 2005; Zayas and Shoda, 2009).

In this study, a dual-path cognitive-affective model grounded in the CAPS theory is used to investigate the impact of CSR perception on employees’ PEB. As described in the CAPS theory, individual cognitive and affective units respond in a specific manner to them (Saleem et al., 2020). The CAPS theory explains changes in individual behavior patterns through the dual pathways of cognition and emotion. It provides an effective theoretical perspective on the influence of perception of CSR on individual complex behaviors and attitudes. First, CSR perception has the potential to trigger cognitive changes in employees that affect their PEB. Meaningful work (MW) can positively influence employees’ attitudes toward work and behavior outside of their regular roles (Sabokro et al., 2021). MW is key to shaping perceptions of CSR and influencing employees’ PEB. Second, CSR perception can have a effect on employees’ PEB through triggering emotional changes in them. In addition to being fundamental employee responses, emotions are also a key mechanism by which work events are translated into employee behavior (Groening and Peloza, 2023). Those employees who feel a sense of connectedness to nature (CN), as a basic human emotion, believe that changes in nature have a direct bearing on their personal fate. As per the CAPS theory, the effectiveness of employees’ cognitions and emotions is determined in large part by the environment in which they work. A green psychological climate (GPC) emerges when employees see that the organization has implemented policies promoting environmental sustainability (Hur et al., 2020).

### 3.2 Hypothesis development

#### 3.2.1 PECSR and PEB

Pro-environmental behavior (PEB) refers to employees’ conscious efforts to reduce or eliminate negative environmental impacts. In addition to positively influencing the natural environment, PEB also encourages others to actively participate in environmental protection activities (Wu et al., 2022). Employees have the option of engaging in environmentally friendly behavior at their discretion. Unless employees identify with the organization’s actions, they will not be able to actively participate in the organization (Story and Castanheira, 2019). PECSR refers to the situational perceptions that employees form when they create change. As it relates to sustainable development (Borland et al., 2018), employees’ own PECSR has a significant impact on their perceptions and intuitions. Meanwhile, employees feel the company’s commitment to environmental protection, which has a significant effect on their psychology (May et al., 2004). In the event that an employee’s emotional processing unit is activated, they are likely to perceive their company as a responsible one, and become emotionally dependent on it as a result. It has been found that when employees perceive the company’s ECSR in a positive light, they will respond positively with their PEB. Based on the above analysis, this study proposes the following hypothesis:

Hypothesis 1 (H1). PECSR is positively related to employees’ PEB.

#### 3.2.2 The moderating role of MW

Marine enterprises confront highly complex, risky, and visible environmental stakes (e.g., oil-spill prevention, ballast-water treatment). Employees therefore need to see a clear line-of-sight between their daily tasks and tangible ecological outcomes. MW is a measure of the goals, tasks, and values associated with work based on personal beliefs and needs (Lysova et al., 2019; Steger et al., 2012). Employees are not only employed to obtain material rewards, but also to gain a sense of self-worth and meaning (the value of the work they perform, the promotion of personal growth, the satisfaction of individual needs, and the positive impact on society) (Autin et al., 2022; Stephan et al., 2020). Under the influence of situational information, individuals are prompted to compare their beliefs and goals to form cognitive needs (Saleem et al., 2020). As a result of addressing individual needs and enhancing cognition, PECSR achieves a significant impact on employees’ sense of MW. The employees feel their efforts and decisions regarding CSR activities are valuable and make a difference. An employee can feel that he or she has sufficient work abilities to complete work tasks, grow at work, and enhance their sense of self-worth at work (Johnson and Jiang, 2017). Employees who engage in continuous cognitive strengthening believe that their work is very important and can positively impact their teams and organizations. This naturally leads to a sense of MW (Allan et al., 2019). In order to foster positive attitudes and behaviors at work, a sense of MW is necessary (Mayer and Frantz, 2004; Schwarz and Clore, 1983). Through PECSR, employees are able to increase their sense of meaning at work through altered perceptions. We propose the following hypotheses:

Hypothesis 2 (H2). MW mediates the relationship between PECSR and employees’ PEB.

#### 3.2.3 The moderating role of CN

While MW captures cognitive evaluation, the marine milieu is also saturated with affectively charged stimuli (open-sea vistas, encounters with marine wildlife). One of the most basic emotional responses of human beings is natural emotional connection. It is possible to categorize an individual’s emotional reaction to nature into two primary categories: a sense of connection, in which one believes one’s interests are intimately associated with the natural environment, and a sense of identity, in which the in dividual and the environment should be intertwined (Robertson, 2018). An individual’s emotions are physiological responses that relate external stimuli to their behavior, and provide an important signal to the environment that enables them to adapt to it (Tam, 2013). CAPS theory holds that an individual’s behavior is largely driven by the activation of emotional units (Saleem et al., 2020). People and nature should live in harmony. A series of CSR activities that focus on environmental issues can enhance the emotional experiences of employees in the most natural and positive manner. Employees will gain a deeper understanding of the relationship between themselves and the environment by participating in environmental protection activities (Bouraoui et al., 2019). Observing the efforts that enterprises make to protect the environment will inspire positive emotional experiences among employees, thereby deepening their CN. As well as promoting employees’ PEB, being connected to nature can also promote their well-being. A connection to nature can also promote employees’ PEB. The development of a deeper emotional bond will also strengthen employees’ commitment to protecting the nature (James et al., 2008). Employees’ involvement in the enterprise’s CSR activities at work, their understanding of the importance of activities, and their emotional CN are factors that motivate them to take part in PEB. Employees’ emotional changes (emotional experiences which strengthen their emotional CN) can trigger their PEB through PECSR. Hypotheses are proposed as follows:

Hypothesis 3 (H3). CN mediates the relationship between PECSR and employees’ PEB.

#### 3.2.4 The dual mediating role of MW and CN

Having a sense of MW refers to evaluating and appreciating the value of work and recognizing one’s own personal growth (Autin et al., 2022). The sense of CN is an individual’s emotional response to the nature (Gkargkavouzi et al., 2018; Robertson, 2018). In accordance with the CAPS theory, the situation in which an individual finds themselves can affect his or her cognitive and affective units, ultimately influencing his or her behavior (Norton et al., 2014). As a result of this theoretical framework, this study assumes that employees’ perceptions of CSR can enhance their feelings of MW and strengthen their emotional CN. In the context of dual stimulation of the cognitive and emotional pathways, employees are likely to exhibit positive PEB. Therefore, we hypothesized that:

Hypothesis 4 (H4). MW and CN provide a dual mediation influence on the relationship between PECSR and employees’ PEB.

#### 3.2.5 The moderating role of GPC

The CAPS theory indicates that situational factors influence a person’s psychological state, which in turn influences their behavior (Norton et al., 2014). An individual’s psychological climate significantly influences their behavior, and employees’ actions are influenced by their perceptions and interpretations of the objective work environment (Tahir et al., 2020). A person’s psychological climate is shaped by their interactions with society. Through these interactions, employees establish their values. The GPC refers to the perception of policies and practices related to environmental sustainability held by employees (Norton et al., 2017; Zacher et al., 2023). Employees with a higher GPC are more environmentally conscious, pay more attention to green development information, and are more supportive of the organization’s efforts (Gosling and Williams, 2010).

By engaging in CSR activities, enterprises convey to their employees the green values that they expect from them. Employees who participate in CSR activities gain a sense of meaning from their work. They feel positive about their enterprise’s green development strategy and are aware that their enterprise must address environmental issues. They also respond positively to their enterprise’s environmental measures in CSR activities. By implementing positive environmental protection behaviors, they contribute to the sustainable development of the enterprise as a result of the enhanced perception of MW. Therefore, the GPC can enhance the positive correlation between MW and PEB. GPC can reflect employees’ understanding and emotional identification with the green goals of the organization (Sabokro et al., 2021). As employees participate in CSR activities and become more aware of the practical application of energy conservation and emissions reduction in the enterprise, their green expectations become clearer to them. Employees with sufficient emotional resources will be more likely to maintain a relaxed state of mind and will gradually become more emotionally connected to the enterprise. By engaging in autonomous PEBs based on their CN, they will be able to contribute to the enterprise and the environment. Consequently, GPC can enhance the positive correlation between CN and PEB. Therefore, it was hypothesized that:

Hypothesis 5 (H5). The GPC moderates the relationship between MW and PEB.

Hypothesis 6 (H6). The GPC moderates the relationship between CN and PEB.

### 3.3 Research model

In this study, the primary objective was to explore the cognitive pathway of perception of CSR-MW-PEB and the emotional pathway of perception of CSR-CN-PEB based on CAPS theory. Furthermore, it aimed to examine the moderating influence of the GPC on the relationship between MW, CN, and PEB. Drawing on the CAPS theory, we argue that a dual-pathway model is indispensable for three reasons. First, environmental CSR messages in marine enterprises are simultaneously laden with rational information (energy-saving targets, regulatory compliance) and emotional cues (ocean conservation narratives). By treating them as parallel mediators, we capture the full bandwidth of employee sense-making and avoid underestimating PECSR’s total effect on PEB. Second, the CAPS framework posits that stable behavioral signatures emerge only when situation-specific cognitive appraisals and affective reactions jointly guide action. Third, partitioning the variance allows us to demonstrate that the two pathways respond differently to contextual strength. Collectively, the dual-pathway lens offers a more granular and holistic account of how environmental CSR translates into employee pro-environmental behavior than single-mechanism models currently dominating the literature.

## 4 Methodology

### 4.1 Population and sampling

This study was conducted on selected employees of marine equipment manufacturing enterprises and marine resource enterprises in eastern China via questionnaires. Our selection of marine enterprises was based primarily on their significant impact on the marine environment through marine pollution, etc., (Ning et al., 2024). In particular, marine enterprises are one of the main contributors to considerable environmental changes in developing countries (Liu et al., 2023; Ye et al., 2022). Further, it has been reported that Chinese marine enterprises are seeking a “green” environment in order to meet the requirements for the development of marine ecological civilization (Deng and Shi, 2023; Jiang et al., 2020; Zhang et al., 2023). Employees in marine enterprises are also better informed of environmental objectives as they are primary stakeholders (Liu et al., 2023). To explore the relationship between PECSR and employees’ PEB, a causal effect research method was adopted. Questionnaires were distributed during non-working hours. Researchers distributed the questionnaires using a major survey platform (Sojump, Chinese name: Wenjuanxing) in China, powered by www.wjx.cn, distributed via WeChat (see Supplementary material) (Chen et al., 2020; Gao et al., 2020; Liu X. et al., 2021; Lu et al., 2021; Montag et al., 2018; Wang et al., 2019; Wu et al., 2023). Respondents voluntarily participated in the survey. The questionnaire survey period is from June 1, 2024 to October 20, 2024, and each participant has obtained informed consent form (Informed consent form is located in the first part of the questionnaire, each participant will see the informed consent form first, and can fill in the questionnaire after agreeing). The main object of this study is the working staff of the enterprise, and minors are not involved. There were 598 questionnaires distributed, and 436 completed questionnaires were retrieved, resulting in a recovery rate of 72.91%. A total of 396 valid questionnaires were ultimately retrieved after removing invalid surveys. The questionnaire’s comprehensive validity percentage was 66.22%. The descriptive statistical analysis of the sample is presented in Table 1.

**TABLE 1:** Respondents’ demographic profile (*N* = 396).

Characteristics	Frequency (f)	Percentage (%)
**Gender**		
Male	230	58.08
Female	166	41.92
**Age**		
21–25	48	12.12
26–5	268	67.68
36–45	71	17.93
>45	9	2.27
**Education**		
High school/junior college	48	12.12
University	311	78.54
Master and above	37	9.34
**Years of service**		
<1	65	16.41
1–5	134	33.84
6–10	88	22.22
11–15	56	14.14
>15	53	13.39
**Enterprise size**		
Small	101	25.50
Medium	124	31.31
Large	171	43.19
**Ownership type**		
State-owned	227	57.32
Private	169	42.68

### 4.2 Measures

To collect quantitative data, the present study utilized the questionnaire survey method with a 5-point Likert scale (see Supplementary material). A score of 5 indicates significant agreement, whereas a score of 1 indicates severe disagreement. A 4-item scale was used to measure PECSR (e.g., “Environmental issues are integral to the strategy of my organization”) (Glavas and Kelley, 2014; Wiefek and Heinitz, 2021), whereas MW employed a 10-item scale (e.g., “I understand how my work contributes to my life’s meaning”) (Fouché et al., 2017; Steger et al., 2012). A 7-item scale was used by CN (such as, “I often feel a part of nature”) (Gosling and Williams, 2010), a 3-item scale was used by PEB (such as “Today, I took initiative to act in environmentally-friendly ways at work”) (Bissing-Olson et al., 2013), and a 5-item scale was applied by GPC (such as, “All employees are encouraged to save energy within the workplace”) (Fang et al., 2022; Sabokro et al., 2021).

## 5 Results

### 5.1 Reliability and validity

The results indicate that the data is good and can be used as a foundation for future analyses. The reliability of all scales was good (Cronbach’s alpha (α) > 0.7) (Hair et al., 2011), as shown in Table 2. In the study, reliability and validity tests were conducted, and the Composite Reliability (CR) values for the variables were 0.857, 0.941, 0.916, 0.873, and 0.916, respectively, indicating greater reliability than the coefficient standard of 0.7; the average variance extracted (AVE) values were 0.599, 0.617, 0.608, 0.697, and 0.686, respectively, higher than the coefficient standard of 0.5 (Alnehabi and Al-Mekhlafi, 2023; Anderson and Gerbing, 1988; Bagozzi and Yi, 1988).

**TABLE 2:** Results of measurement model.

Constructs	α	AVE	CR
PECSR	0.855	0.599	0.857
MW	0.941	0.617	0.941
CN	0.915	0.608	0.916
PEB	0.872	0.697	0.873
GPC	0.915	0.686	0.916

The study employed AMOS 23.0 software for validated factor analysis, constructing one-factor, two-factor, three-factor, four-factor, and five-factor structural models in order to assess the discriminant validity of latent variables and the fit of the model. Using confirmatory factor analysis (CFA), multiple factor analysis models were also evaluated to validate the factor structure and comparison (Liu et al., 2023). Presented in Table 3, the results indicate that the five-factor model exhibits an optimal fit, and the discriminant validity among the five variables is superior, facilitating the subsequent analysis phase.

**TABLE 3:** Model comparison using CFA.

Model	*X^2^/df*	CFI	TLI	NFI	GFI	RMSER
Five-factor model	1.260	0.987	0.986	0.940	0.926	0.026
Four-factor model	5.091	0.792	0.773	0.755	0.623	0.102
Three-factor model	6.404	0.723	0.700	0.690	0.559	0.117
Two-factor model	9.891	0.543	0.506	0.518	0.474	0.150
Single-factor model	11.167	0.476	0.435	0.454	0.430	0.160

CFI, comparative fit index; TLI, tucker-lewis index; NFI, normed fit index; IFI, incremental fit index; SRMR, standardized root-mean-square residual.

### 5.2 Common method bias (CMB)

In view of the fact that the study questionnaire utilized a self-assessment method, CMB is possible among the variables. Therefore, CMB was examined during the data inspection process (Ali et al., 2021; Conway and Lance, 2010; Podsakoff et al., 2003; Spector, 2006). To verify the data, Harman one-factor analysis was used (Aguirre-Urreta and Hu, 2019; Fuller et al., 2016; Howard et al., 2024; Howard, 2023). As a result of the test, the variance of the first main component accounted for 32.86%, which falls short of the 40% threshold. An analysis of the variables using a one-factor confirmatory factor analysis showed that the fit is poor (X2/df = 11.167, CFI = 0.476, TLI = 0.435, NFI = 0.454, GFI = 0.430, RMSER = 0.160). It can be concluded from the above results that this study has a better control of CMB.

### 5.3 Correlation

The study tested the means, standard deviations, and correlation coefficients of the variables, and the results are shown in Table 4. PECSR was positively correlated with positive environmental behavior (*r* = 0.515, *p* < 0.01); PECSR was associated with sense of meaning at work and natural emotional connection (*r* = 0.378, *p* < 0.01; *r* = 0.442, *p* < 0.01); and sense of meaningfulness at work and natural emotional connection were positively correlated with positive environmental behavior (*r* = 0.423, *p* < 0.01; *r* = 0.467, *p* < 0.01). The correlations of the main variables were in line with expectations, and the preliminary test results supported the previous hypotheses.

**TABLE 4:** Correlation matrix.

Constructs	Mean	S.D	1	2	3	4	5
1 PECSR	3.592	0.872	1.000				
2 MW	3.329	0.895	0.378**	1.000			
3 CN	3.558	0.861	0.442**	0.347**	1.000		
4 PEB	3.449	1.027	0.515**	0.423**	0.467**	1.000	
5 GPC	3.168	1.015	−0.040	0.070	−0.034	0.044	1.000

S.D, standard deviation;

** denotes *p* < 0.01.

### 5.4 Model adequacy

**FIGURE 1:**
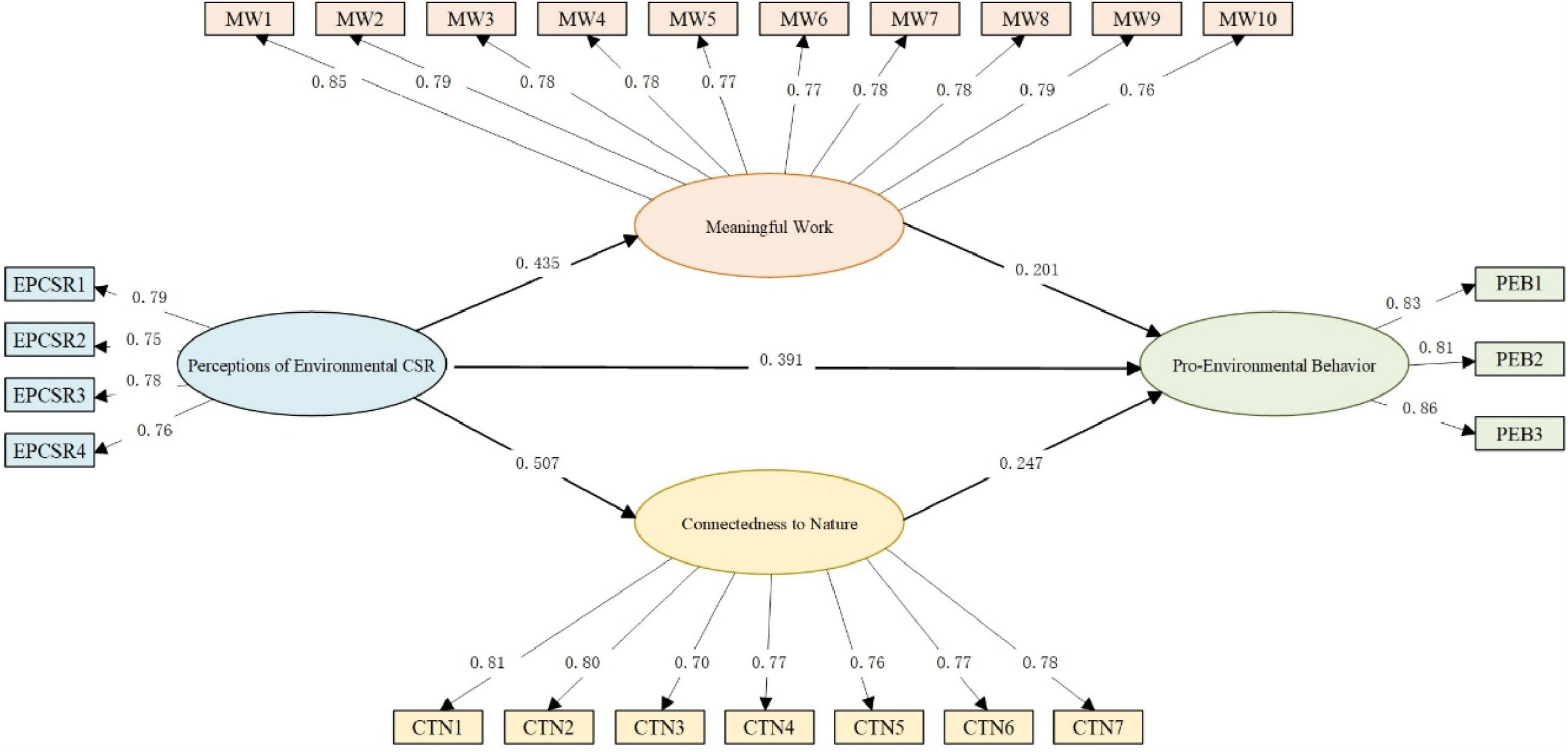
SEM algorithm results of proposed model.

As shown in Figure 1, the hypothesis test findings of the structural equation model (SEM) indicate that a significant path coefficient GPC from PECSR to PEB (β = 0.391, *p* < 0.01), confirming that PECSR is significantly positively associated with PEB and validating Hypothesis H1. The test results indicate that PECSR covaries positively with MW (β = 0.435, *p* < 0.01), while MW significantly predicts PEB (β = 0.201, *p* < 0.01). MW also covaries positively with CN (β = 0.507, *p* < 0.01), and CN is similarly linked to PEB (β = 0.247, *p* < 0.01). The aforementioned test results establish the foundation for the examination of mediating effects.

There are two pathways through which PECSR influences PEB: cognitive and affective, and the test results indicate that the model fits well overall (X2/df = 2.201, CFI = 0.951, NFI = 0.914, GFI = 0.951, RMSER = 0.055). Table 5 shows the results of a further double mediation effect test. It was determined that the mediating effect is significant for the cognitive pathway “PECSR → MW → PEB” with an effect of 0.092, a 95% confidence interval of [0.049, 0.156], not including 0, indicating a significant effect for the mediating effect, and H2 was further verified. In the affective pathway “PECSR→CN→PEB”, the effect value is 0.132, the 95% confidence interval is [0.076, 0.204], not including 0, indicating that the mediating effect is significant, and H3 is also confirmed. In addition, the test results confirm that PECSR influences PEB through both cognitive and affective pathways, and that the MW and CN play a dual mediated role. It has been verified that H4 is correct.

**TABLE 5:** Pathway coefficients of the model.

Variable relationship	Effect	LLCI	ULCI
PECSR MW → PEB	0.092	0.049	0.156
PECSR → CN → PEB	0.132	0.076	0.204
Median effect value	0.225	0.154	0.311

LLCI, lower limit confidence interval; ULCI, upper limit confidence interval.

**FIGURE 2:**
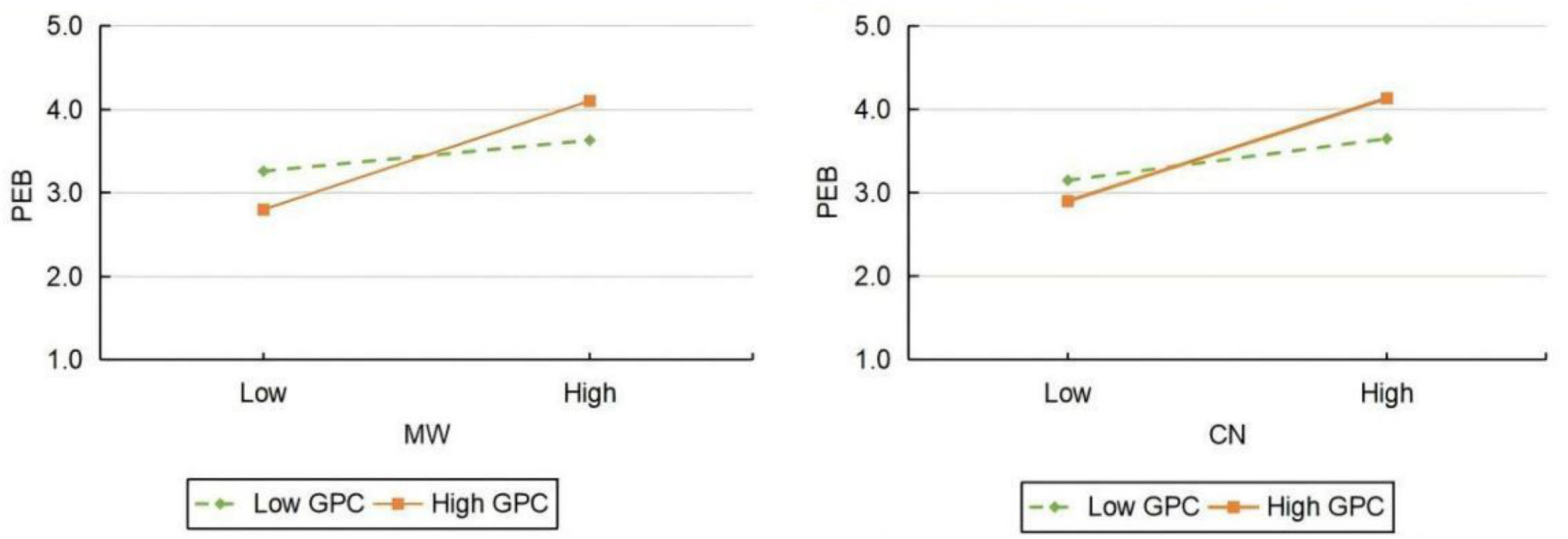
Simple slope analysis.

A moderated mediation test confirmed the moderating effect of GPC in the MW and PEB as well as the moderating effect of GPC in the CN and PEB. MW significance and GPC interacted significantly to affect positive PEB (β = 0.241, *p* < 0.01), with a 95% confidence interval of [0.147, 0.335], not including 0. GPC acted as a positive moderator between MW and PEB, and H5 was found to be valid. There is an interaction term between CN and GPC that affects the positive PEB (β = 0.210, *p* < 0.01), with a 95% confidence interval of [0.107, 0.313], not including 0. Accordingly, GPC plays a positive moderating role between CN and PEB, and H6 is confirmed. It has been found that the positive effect of MW on PEB is more significant when the level of GPC is higher, based on the analysis of the process plug-in moderating effect. A higher GPC level results in a stronger positive effect of CN on PEB. In order to further assess the moderating effect, this study used high (mean + standard deviation) and low (mean - standard deviation) GPC as grouping variables to plot the moderating effect, as shown in Figure 2.

## 6 Discussion

**FIGURE 3:**
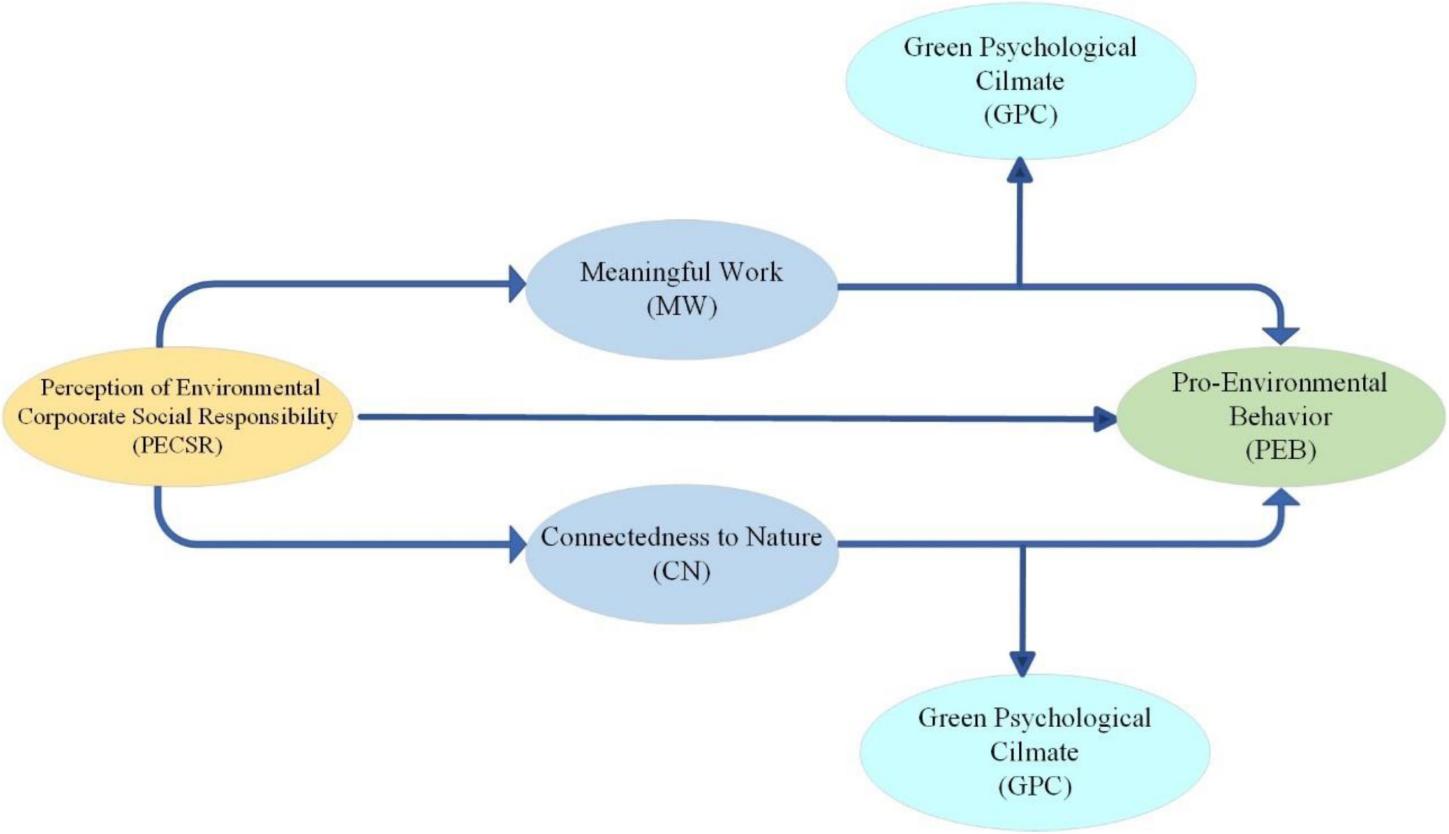
Research model.

A number of academicians and policymakers are interested in the topic of CSR perceptions. Past studies have also examined the relationship between CSR perceptions and employee behavior. However, the research on PECSR has been less involved (Abbas and Dogan, 2022; Aukhoon et al., 2024; Suganthi, 2019). In addition, a number of studies have investigated the relationship between CSR and employees’ environmental behavior in hospitals, hotels, and manufacturing, but there is a dearth of research on marine enterprises (Bai et al., 2024; Deng et al., 2022a; Liu et al., 2023). Thus, this study developed a theoretical research model based on data from Chinese marine enterprises to examine how PECSR impacts employees’ PEB in accordance with the CAPS theory,as shown in Figure 3. As a result of dual-pathway effects of MW and CN, PECSR is found to positively influence employees’ PEB. The GPC is intended to enhance the influence of MW and CN on the PEB of employees.

First, the effect of PECSR on employees’ PEB is examined. An organization’s CSR activities related to the protection of the environment have a positive effect on its employees’ environmental perceptions. Employee perceptions have a significant impact on employee behavior, which in turn affects the development of the business. MW and CN were found to play a mediating role in the study. There is consistency in this finding with previous studies (Wang et al., 2022), which suggest that PECSR plays an important mediating role and enhances the influence of PECSR on employees’ PEB. Second, PECSR affects employee behavior from both cognitive and affective pathways, and employees’ positive perceptions of and emotional resonance with CSR activities will contribute greatly to their PEB. By verifying the dual-pathway roles of MW and CN, this study clarifies the transmission mechanism between PECSR and employees’ PEB in a more systematic and complete manner. Revealing the cognitive and affective processes through which PECSR affects PEB and developing a deeper understanding of the reasons behind employees’ PEB implementation. Moreover, it provides a new theoretical perspective for explaining the mechanism of action of PECSR. Third, the boundary role of GPC was examined. The GPC, as a contextual factor, plays a positive moderating role between PECSR and PEB. The psychological climate of employees is an important predictor of individual behavior, and employees’ behaviors are influenced by how they perceive and interpret the objective work environment (James et al., 2008). Employees who participate in CSR activities have a positive impact on the organization’s green development strategy.

Employees’ perceptions and interpretations of organizational policies, as well as the rewards and support measures adopted by the organization, all subtly shape the employees’ GPC. This is reflected in their understanding and feelings toward the green policies, philosophies, and objectives implemented by the organization. GPC not only reflects the extent of employees’ recognition and support for the environmental initiatives undertaken by the organization, but also determines their behavior and attitudes at work to a certain extent. Existing research has confirmed that employees’ perceptions of GPC can influence their environmental behavior (Dumont et al., 2017; Norton et al., 2014).

### 6.1 Theoretical implications

This study advances theoretical understanding in three key areas. First, it extends CAPS theory by empirically validating PECSR as a critical contextual antecedent activating employees’ cognitive-affective pathways to drive PEB. This addresses the gap in CSR literature regarding how PECSR translate into employee behaviors. Second, we resolve theoretical ambiguities about MW and CN to a certain degree. The study of MW is still in its infancy, and some scholars have proposed that the triggering factor of MW is individual traits, and that MW can enhance an employee’s commitment to his or her job (Autin et al., 2022; Song et al., 2023). The study confirms that the significance of MW is affected by PECSR and promotes employees’ PEB, as well as introducing a GPC to further explain the significance of MW within the boundary, in response to the scholars’ appeal (Norton et al., 2014). For CN—a construct underexplored in management—we establish its role as an affective mechanism linking CSR to PEB, broadening the range of research and application contexts for the concept. Third, we introduce that GPC has a moderating effect on employees’ PEB, which helps to deepen their understanding of the phenomenon. Our moderated-mediation model reveals GPC amplifies the PECSR→MW/CN→PEB pathway, underscoring organizational climate’s role in activating CSR’s cognitive-affective effects. CSR activities can enhance the impact of the contextual climate on employees by improving transparency and increasing implementation frequency, thus promoting PEB through the enhancement of GPC.

### 6.2 Practical implications

To effectively translate CSR into employees’ PEB, organizations—particularly in the marine sector—must strategically leverage the pathways identified in this study. Firstly, enhance PECSR through transparent and frequent communication of specific environmental initiatives (e.g., waste reduction in ports, sustainable fishery practices), moving beyond generic statements to demonstrate tangible commitment and combat skepticism. Secondly, actively cultivate the mediating states: Design CSR activities (e.g., coastal clean-ups, coral restoration volunteering) to intrinsically foster employees’ sense of MW by connecting their roles to environmental impact, and deepen CN through immersive experiences relevant to marine environments. Thirdly, deliberately shape the GPC: Integrate sustainability visibly into daily operations (e.g., using eco-materials onboard/in offices, implementing energy-saving measures) and train managers to model and endorse PEB, thereby amplifying the positive effects of PECSR, MW, and CN. By embedding these strategies—focused on credible PECSR, enhanced MW/CN, and a strong GPC—into core management practices and sustainability audits, enterprises can transform employee environmental consciousness into sustained action, ultimately achieving both ecological and organizational sustainability goals.

### 6.3 Limitations and future research directions

While the study offers fresh theoretical and managerial insights, it is bounded by several inter-related limitations that future research should systematically address. First, the sample’s marine enterprises focus limits external validity; multi-industry extensions using stratified or meta-analytic designs are required. Second, reliance on Harman’s single-factor test alone is insufficient to rule out common method bias inherent in self-reported data; follow-up studies should complement this with CFA marker-variable techniques, multi-source (supervisor/HR) or multi-level (team climate) data, and procedural remedies such as temporal or psychological separation of measures. Third, the cross-sectional design precludes causal inference; longitudinal panels, field or quasi-experiments, and experience-sampling methods are recommended to capture temporal ordering and within-person dynamics.

## 7 Conclusion

Using Chinese marine enterprises as a research sample, this study examines how PECSR impacts employees’ PEB according to the CAPS theory by considering the mediating roles of the MW, CN, and the GPC. In the present study, we found that PECSR was significantly positively associated with PEB, with MW and CN acting as a bridging mechanism, and a GPC supporting the positive effect. In this study, PECSR was found to be an important factor in CSR. When planning sustainability activities for targeted strategy development, enterprises should take this influence into consideration.

## Data Availability

The original contributions presented in this study are included in this article/Supplementary material, further inquiries can be directed to the corresponding author.
