# Supplementary material for: Unveiling the dual-pathway effect of CSR perception environmental on employee pro-environmental behavior: evidence from Chinese marine enterprises
Source: Front Psychol. 2025 Aug 25;16:1649048. doi: 10.3389/fpsyg.2025.1649048 (PMC12415001; doi:10.3389/fpsyg.2025.1649048)
Supplement: Supplementary file 1 [file Supplementary_file_1.docx]

Supplementary material

A. Questionnaire

**企业环保活动对员工行为影响的问卷调查**

尊敬的先生/女士您好：
   本问卷旨在了解您所在公司环保活动对您的行为影响的一些情况。本研究仅用于学术研究，所有内容将予以严格保密，恳请您仔细阅读并根据项目实际情况放心填写。每个问题所反映的信息都对我们非常珍贵，问题的回答没有对与错之分。感谢您对我们科研工作的支持，衷心祝您工作顺利。

您的性别： ○男 ○女

您的年龄: ○21~25 ○26~35 ○36~45 ○45以上

您的学历：○高中/大专 ○大学本科 ○研究生及以上

您的工作年限: ○不足1年 ○1-5年 ○6-10年 ○11-15年 ○15年以上

您所在企业的规模: ○小型企业（1-100人） ○中型企业（101-500人） ○大型企业（501人以上）

您所在企业的性质: ○国有企业 ○民营企业

1. 环境问题是我们公司战略的组成部分。

很不同意 ○1 ○2 ○3 ○4 ○5 很同意

1. 处理环境问题是本公司日常运作不可或缺的一部分。

很不同意 ○1 ○2 ○3 ○4 ○5 很同意

1. 我的公司非常注意我们的工作不损害环境。

很不同意 ○1 ○2 ○3 ○4 ○5 很同意

1. 我的公司在关注其对环境影响的同时实现了其短期目标。

很不同意 ○1 ○2 ○3 ○4 ○5 很同意

1. 我找到了一份有意义的工作。

很不同意 ○1 ○2 ○3 ○4 ○5 很同意

1. 我认为我的工作有助于我的个人成长。

很不同意 ○1 ○2 ○3 ○4 ○5 很同意

1. 我的工作对世界有影响。

很不同意 ○1 ○2 ○3 ○4 ○5 很同意

1. 我明白我的工作对我人生意义的贡献。

很不同意 ○1 ○2 ○3 ○4 ○5 很同意

1. 我很清楚是什么让我的工作有意义。

很不同意 ○1 ○2 ○3 ○4 ○5 很同意

1. 我知道我的工作给世界带来了积极的影响。

很不同意 ○1 ○2 ○3 ○4 ○5 很同意

1. 我的工作帮助我更好地了解自己。

很不同意 ○1 ○2 ○3 ○4 ○5 很同意

1. 我已经找到了有令人满意目的的工作。

很不同意 ○1 ○2 ○3 ○4 ○5 很同意

1. 我的工作帮助我理解周围的世界。

很不同意 ○1 ○2 ○3 ○4 ○5 很同意

1. 我的工作有更大的意义。

很不同意 ○1 ○2 ○3 ○4 ○5 很同意

1. 我常常觉得自己是大自然的一部分。

很不同意 ○1 ○2 ○3 ○4 ○5 很同意

1. 我感到与自然世界很亲近。

很不同意 ○1 ○2 ○3 ○4 ○5 很同意

1. 我与周围的自然环境感觉到有联结。

很不同意 ○1 ○2 ○3 ○4 ○5 很同意

1. 我不会感到与大自然脱节。

很不同意 ○1 ○2 ○3 ○4 ○5 很同意

1. 我自己的幸福与自然界的幸福是联系在一起的。

很不同意 ○1 ○2 ○3 ○4 ○5 很同意

1. 我认为万物皆有灵。

很不同意 ○1 ○2 ○3 ○4 ○5 很同意

1. 我认为所有自然生物都是平等的。

很不同意 ○1 ○2 ○3 ○4 ○5 很同意

1. 我会抓住机会在工作中积极参与环保工作。

很不同意 ○1 ○2 ○3 ○4 ○5 很同意

1. 我在工作中会主动采取环保行动。

很不同意 ○1 ○2 ○3 ○4 ○5 很同意

1. 我为工作环境所做的比我预期的要多。

很不同意 ○1 ○2 ○3 ○4 ○5 很同意

1. 我的公司鼓励所有员工在工作场所节约能源。

很不同意 ○1 ○2 ○3 ○4 ○5 很同意

1. 我的公司在生产过程中强调减少废料。

很不同意 ○1 ○2 ○3 ○4 ○5 很同意

1. 我的公司已经公布了工作场所的环境政策。

很不同意 ○1 ○2 ○3 ○4 ○5 很同意

1. 我的公司的管理和政策有环境保护措施。

很不同意 ○1 ○2 ○3 ○4 ○5 很同意

1. 我的公司领导有减少浪费和控制有害化学物质的行为。

很不同意 ○1 ○2 ○3 ○4 ○5 很同意

B. Scale

|  |  |
| --- | --- |
| PECSR | Environmental issues are integral to the strategy of my organization  Addressing environmental issues is integral to the daily operations of my organization  My organization takes great care that our work does not hurt the environment  My organization achieves its short-term goals while staying focused on its impact on the environment |
| MW | I have found a meaningful career  I view my work as contributing to my personal growth.  My work really makes difference to the world.  I understand how my work contributes to my life’s meaning.  I have a good sense of what makes my job meaningful.  I know my work makes a positive difference in the world.  My work helps me better understand myself.  I have discovered work that has a satisfying purpose.  My work helps me make sense of the world around me.  The work I do serves a greater purpose. |
| CN | I often feel that I am a part of nature.  I often feel close to the natural world around me.  I never feel a personal bond with things in my natural surroundings like trees, wildlife or the view on the horizon.  I often feel connected from nature.  My own welfare is linked to the welfare of the natural world.  I recognise and appreciate the intelligence of other living things.  When I think about my place on the planet, I don’t consider myself to be at the top of  a pecking order among all living things. |
| PEB | Today, I took a chance to get actively involved in environmental protection at work.  Today, I took initiative to act in environmentally-friendly ways at work.  Today, I did more for the environment at work than I was expected to. |
| GPC | All employees are encouraged to save energy within the workplace.  There is an emphasis on the reduction of scraps during production.  The company has announced the general environmental policies at the workplace.  Company management and policies lead to environmental preservation.  Company managers try to reduce wastes and control harmful chemicals. |
